# Type 2 diabetes mellitus burdens among adults with sickle cell disease: A 12‐year single health system‐based cohort analysis

**DOI:** 10.1002/jha2.161

**Published:** 2021-01-04

**Authors:** Jifang Zhou, Gregory S. Calip, Edith A. Nutescu, Jin Han, Surrey M. Walton, Andrew Srisuwananukorn, William L. Galanter

**Affiliations:** ^1^ School of International Pharmaceutical Business China Pharmaceutical University Nanjing China; ^2^ Center for Pharmacoepidemiology and Pharmacoeconomic Research University of Illinois at Chicago Chicago Illinois USA; ^3^ Department of Pharmacy Systems, Outcomes and Policy, College of Pharmacy University of Illinois at Chicago Chicago Illinois USA; ^4^ Flatiron Health New York New York USA; ^5^ Department of Pharmacy Practice, College of Pharmacy University of Illinois at Chicago Chicago Illinois USA; ^6^ Division of Hematology & Oncology, Department of Medicine, Comprehensive Sickle Cell Center University of Illinois at Chicago Chicago Illinois USA; ^7^ Section of Academic Internal Medicine & Geriatrics Department of Medicine University of Illinois at Chicago Chicago Illinois USA

**To the Editor**:

Recent observational studies have demonstrated similar type 2 diabetes mellitus (T2DM) prevalence in commercially insured sickle cell disease (SCD) patients compared with the general population.[1, 2] However, several important questions about T2DM in SCD remain unanswered. Past studies are limited due to lack of definitive genotype information [3, 4, 5]; it is possible that different SCD genotypes (sickle cell anemia (HbSS), sickle hemoglobin C disease (HbSC), sickle β^+^‐thalassemia (HbSβ^+^), and sickle β^0^‐thalassemia (HbSβ^0^)) demonstrate varying risks of T2DM and its associated complications. Additionally, important information on anthropometrics were lacking in most epidemiology studies. It is unclear whether low lean body weight and fat mass in anthropomorphic studies associated with SCD conferred protection against T2DM [6, 7].

In this single health‐system based, retrospective cohort study, we extracted 12 years (2008–2019) of electronic health records (EHR) data from a large, urban, tertiary health care system. Patients who self‐identified as African American (AA) were included in the analysis. SCD genotypes were ascertained through a combination of chart review and by hemoglobin electrophoresis test records extracted from EHR. Identified T2DM cases with and without SCD were then matched by exact sex and age in up to a 1:10 ratio. The Institutional Review Board of the University of Illinois at Chicago approved this study. Categorical variables were compared using the chi‐square test or Fisher's exact test, while continuous measurements between groups were assessed using the nonparametric Wilcoxon rank sum test to account for nonnormal distribution of the variables such as age and body mass index (BMI). Multivariable logistic regression analysis was used to assess the association between T2DM and SCD status, after accounting for sex, age, BMI, insurance type, and household income levels. A marginal multilevel model was used, with SCD status as a between‐subject effect, and individual plasma glucose values as repeated measures [8]. All analyses were conducted using SAS 9.4 (Cary, North Carolina) and a two‐tailed *P* value of less than 0.05 was used to determine statistical significance.

T2DM was identified in 89 out of the 634 patients with SCD and aged ≥ 20 years old. Among these patients, mean [median] age was greater among SCD patients with T2DM compared to SCD patients without T2DM (43.3 [44] vs. 32.6 [29] years; *p *< 0.01). SCD patients with T2DM were found to have higher mean [median] BMI compared to SCD patients without SCD (28.9 [27.8] vs. 25.5 [24.0]; *p *< 0.01). Patients with SCD had lower BMI compared to age‐ and sex‐matched AAs with T2DM (28.9 [27.8] vs. 36.7 [35.3]; *p *< 0.01). Among 89 patients with SCD and T2DM, 22 (24.7%) were on treatment for diabetes (Table 1 and Table S1).

**TABLE 1:** Descriptive characteristics of sickle cell disease patients with and without T2DM, 2008–2019

	SCD with T2DM (*n* = 89)		SCD without T2DM (*n* = 545)		
	N	(%)	N	(%)	*p* ^a^
Age, y					
Median (IQR)	44 (32‐54)		29 (23‐40)		<.01
Mean (SD)	43.3 (13.6)		32.6 (11.4)		
20–29	20	22.5%	287	52.7%	<.01
30–39	12	13.5%	116	21.3%	
40–49	24	27.0%	89	16.3%	
50–59	22	24.7%	41	7.5%	
60–69	10	11.2%	9	1.7%	
70+	1	1.1%	3	0.6%	
Index year					
2008–2010	60	67.4%	304	55.8%	.07
2011–2013	17	19.1%	100	18.3%	
2014–2016	7	7.9%	95	17.4%	
2017–2019	5	5.6%	46	8.4%	
Sex					
Male	29	32.6%	220	40.4%	.16
Female	60	67.4%	325	59.6%	
BMI					
Mean (SD)	28.9 (7.4)		25.5 (6.8)		
Median (IQR)	27.8 (23,1‐32.8)		24.0 (21.3‐27.6)		<.01
Charlson comorbidity score					
0	2	2.2%	247	45.3%	<.01
1	13	14.6%	119	21.8%	
2	9	10.1%	65	11.9%	
3+	65	73.0%	114	20.9%	
Household incomes*					
$1 under $25,000	6	6.7%	25	4.6%	.60
$25,000 under $50,000	61	68.5%	346	63.5%	
$50,000 under $75,000	18	20.2%	137	25.1%	
$75,000 under $100,000	4	4.5%	33	6.1%	
v$100,000 under $200,000	0	0.0%	4	0.7%	
v$200,000 or more	6	6.7%	25	4.6%	
Insurance type					
Commercial	76	85.5%	426	78.1%	.10
Medicaid	11	12.4%	107	19.6%	
Medicare	2	2.2%	11	2.0%	
Self‐pay	0	0.0%	1	0.2%	

Abbreviations: BMI, body mass index; SCD, sickle cell disease; T2DM, type 2 diabetes mellitus.

* Based on Zip Code Characteristics: Median Household Income using the American Community Survey (ACS) providing characteristics at the zip code level with the release of the first 5‐year product (2005–2009).

The types of comorbidities differed by SCD status in the T2DM population, and SCD patients also were at a greater risk of SCD‐related comorbidities. A higher proportion of SCD patients with T2DM developed diabetic nephropathy (44.9% vs. 12.0%; *p *< 0.01), peripheral circulatory complications (18.0% vs. 12.0%; *p *= 0.11), foot ulcer (12.4% vs. 3.4%; *p *< 0.01), as well as myocardial infarction (13.5% vs. 5.8%; *p *< 0.01) (Table 2). After standardization to the 2010 US Census of AA population, the prevalence rates of T2DM were 14.5%, 18.9%, and 16.8% for HbSS, HbSC, and HbSβ^+^ thalassemia patients (Table S2).

**TABLE 2:** Demographic and clinical characteristics of sickle cell disease patients by genotype, 2008–2019

	SCDw/ T2DM (*n* = 89)		Unmatched African Americans w/T2DM (*n* = 9950)							
	N	(%)	N	(%)	*p‐*Value	SCDw/ T2DM (*n* = 89)		Matched African Americans w/T2DM (*n* = 856)		*p‐*Value
Age, y										
Mean (SD)	43.3 (13.6)		54.5 (13.9)			43.3	13.6	44.1	13.1	
Median (IQR))	44 (32‐54)		55 (46‐64)		<0.01	44	32–54	44	34–54	.54
Sex										
Male	29	32.6%	3566	35.8%	.52	29	32.6%	262	30.6%	.70
Female	60	67.4%	6384	64.2%		60	67.4%	594	69.4%	
BMI										
Mean (SD)	28.9 (7.4)		35.2 (10.4)			28.9	7.4	36.7	10.1	
Median (IQR)	27.8 (23.1‐32.8)		33.4 (28.3‐40.2)		<.01	27.8	23.1–32.8	27.8	23.1–32.8	<.01
Charlson comorbidity score										
0	2	2.2%	297	3.0%	.09	2	2.2%	65	7.6%	.12
1	13	14.6%	743	7.5%		13	14.6%	107	12.5%	
2	9	10.1%	1070	10.8%		9	10.1%	130	15.2%	
3+	65	73.0%	7840	78.8%		65	73.0%	554	64.7%	
Type 2 diabetes										
Hypertension	79	88.8%	8116	81.6%	.08	79	88.8%	648	75.7%	<.01
Dyslipidemia	47	52.8%	5858	58.9%	.25	47	52.8%	476	55.6%	.61
Chronic obstructive pulmonary disease	51	57.3%	3052	30.7%	<.01	51	57.3%	305	35.6%	<.01
Vaso‐occlusive crisis	68	76.4%	6	0.1%	<.01	68	76.4%	1	0.1%	<.01
Chronic renal disease	41	46.1%	1896	19.1%	<.01	41	46.1%	133	15.5%	<.01
Acute chest syndrome	29	32.6%	1	0.0%	<.01	29	32.6%	0	0.0%	<.01
Pulmonary hypertension	48	53.9%	563	5.7%	<.01	48	53.9%	46	5.4%	<.01
Stroke/transient ischemic attack	32	36.0%	856	8.6%	<.01	32	36.0%	56	6.5%	<.01
Iron overload	14	15.7%	18	0.2%	<.01	14	15.7%	1	0.1%	<.01
Avascular necrosis	29	32.6%	92	0.9%	<.01	29	32.6%	12	1.4%	<.01
Splenomegaly	4	4.5%	128	1.3%	<.01	4	4.5%	15	1.8%	.08
Splenic sequestration	2	2.2%	0	0.0%	<.01	2	2.2%	0	0.0%	<.01
Hypersplenism	0	0.0%	2	0.0%	.89	0	0.0%	0	0.0%	N/A
T2DM‐related microangiopathy										
T2DM nephropathy	40	44.9%	1571	15.8%	<.01	40	44.9%	103	12.0%	<.01
Peripheral neuropathy	23	25.8%	1773	17.8%	.05	23	25.8%	161	18.8%	.11
Ophthalmic complications	12	13.5%	2139	21.5%	.07	12	13.5%	171	20.0%	.14
T2DM‐related macroangiopathy										
Peripheral circulatory complications	16	18.0%	1572	15.8%	.58	16	18.0%	103	12.0%	.11
Foot ulcer	11	12.4%	344	3.5%	<.01	11	12.4%	29	3.4%	<.01
Amputation	0	0.0%	22	0.2%	.66	0	0.0%	0	0.0%	N/A
Myocardial infarction	12	13.5%	560	5.6%	<.01	12	13.5%	50	5.8%	<.01
Metabolic diabetic complications	1	1.1%	157	1.6%	.73	1	1.1%	14	1.6%	.71

Abbreviations: N/A, not applicable; SCD, sickle cell disease; T2DM, type 2 diabetes mellitus.

SCD patients were found to have comparable risk for T2DM relative to non‐SCD self‐identified AA patients (odd ratio [OR] 1.01, 95%CI (0.79‐1.27), after accounting for age, sex, BMI, and household income and insurance plan types (Table S3). SCD patients with T2DM and valid laboratory results on plasma glucose were found to have lower glucose levels compared to age‐ and sex‐matched AA subjects with T2DM (average glucose levels, 108.6 mg/dL vs. 132.2 mg/dL, estimated difference [standard error]: −17.30 [4.38] mg/dL) (Table S4).

This is the first study examining the association of BMI with T2DM occurrence in patients with SCD. The T2DM risk was comparable after adjusting for demographic, socioeconomic factors, and most importantly BMI. Furthermore, our results demonstrated that patients with HbSS subtype had lower BMI and earlier onset of T2DM. It is possible that depletion of susceptible patients with SCD due to life‐threatening end organ damage could have attributed the early onset of T2DM, as patients were less likely to live through to elderly adulthood and develop T2DM as patients with HbSC and HbSβ^+^ thalassemia. Moreover, our findings support that milder forms of SCD not only had comparable BMI to the general population of AAs, but also share similar T2DM‐related disease characteristics followed at the same institution.

It is worth noting that we identified higher rates of T2DM‐related complications compared to our previous study using nationally representative sample of commercially insured SCD patients. Given that SCD alone can cause end organ damage, additional diabetic complications could accelerate functional decline of the kidney, lung, and central nervous system [9]. Mechanistic studies demonstrated that endothelial injury could have contributed to the development of acute myocardial infarction and unstable angina [10]. Our findings were based on records from a tertiary health system; therefore, it may represent a sicker group of patients who required medical attention in the first place. AA males have lower likelihood visiting healthcare facilities due to complex reasons, and in our identified patient population [11], underrepresentation could undermine the accuracy in estimating the overall T2DM burden. During the COVID‐19 pandemic, individuals with SCD are experiencing even more diagnostic, treatment, and logistical challenges in meeting the healthcare needs.

A fundamental strength of this study is the identification of the cohort through a combination of inpatient, outpatient, laboratory, and pharmacy records from a 12‐year span. Furthermore, we conducted medical chart review and cross‐validation for key patient features such as SCD genotypes and BMI. While such an approach allowed an effective comparison of SCD with thousands of general patients sharing similar characteristics and followed at the same institution, these records are often incomplete and may be subject to suboptimal coding quality.

Overall, these results are consistent with our previous findings using a national claims database, the elevated risks for both T2DM‐related microvascular and macrovascular complications warrant further investigation.

## CONFLICT OF INTEREST

Dr. Calip reports current employment with Flatiron Health, Inc., which is an independent subsidiary of the Roche group. No other authors have disclosures to report.

## AUTHOR CONTRIBUTIONS

J.Z., G.S.C., J.H., and W.L.G. designed and performed the research study. J.Z. analyzed the data. All authors contributed to the drafting, revision, and final approval of the manuscript.

## Supporting information

Supporting information

## Data Availability

The data that support the findings of this study are available from the corresponding author upon reasonable request.
