# Supplementary material for: Type 2 diabetes mellitus burdens among adults with sickle cell disease: A 12‐year single health system‐based cohort analysis
Source: EJHaem. 2021 Jan 4;2(1):94–8. doi: 10.1002/jha2.161 (PMC9175929; doi:10.1002/jha2.161)
Supplement: Supplementary file 1 — Supporting information [file JHA2-2-94-s001.docx]

**Title:** Type 2 Diabetes Mellitus Burdens in Adult Patients with Sickle Cell Disease living in Metropolitan Chicago Area: A Retrospective 12-year Cohort Study

Supplemental material

| **Supplemental Table 1.** Descriptive characteristics of patients by sickle cell disease status, 2008-2019 | | | | | |
| --- | --- | --- | --- | --- | --- |
|  | **SCD**  **(n=634)** | | **Non-SCD**  **African Americans**  **(n=39,137)** | | *P*^a^ |
|  | n | (%) | n | (%) |  |
| **Age, y** |  |  |  |  |  |
| Median (interquartile range) | 30 | 24-43 | 47 | 33-59 |  |
| Mean (SD) | 34.09 | 12.33 | 46.70 | 16.45 | <0.01 |
| 20-29 | 307 | 48.4% | 7630 | 19.5% | <0.01 |
| 30-39 | 128 | 20.2% | 6752 | 17.3% | <0.01 |
| 40-49 | 113 | 17.8% | 7182 | 18.4% |  |
| 50-59 | 63 | 9.9% | 8537 | 21.8% |  |
| 60-69 | 19 | 3.0% | 5537 | 14.1% |  |
| 70+ | 4 | 0.6% | 3499 | 8.9% |  |
| **Index year** |  |  |  |  |  |
| 2008-2010 | 364 | 57.4% | 16973 | 43.4% | <0.01 |
| 2011-2013 | 117 | 18.5% | 7963 | 20.3% |  |
| 2014-2016 | 102 | 16.1% | 9751 | 24.9% |  |
| 2017-2019 | 51 | 8.0% | 4450 | 11.4% |  |
| **Sex** |  |  |  |  |  |
| Male | 249 | 39.3% | 12154 | 31.1% | <0.01 |
| Female | 385 | 60.7% | 26983 | 68.9% |  |
| **Charlson comorbidity score** |  |  |  |  |  |
| 0 | 249 | 39.3% | 10243 | 26.2% | <0.01 |
| 1 | 132 | 20.8% | 6013 | 15.4% |  |
| 2 | 74 | 11.7% | 5356 | 13.7% |  |
| 3+ | 179 | 28.2% | 17525 | 44.8% |  |
| **Household incomes*** |  |  |  |  |  |
| 1 = $1 under $25,000 | 31 | 4.9% | 3391 | 8.7% | <0.01 |
| 2 = $25,000 under $50,000 | 407 | 64.2% | 27747 | 70.9% |  |
| 3 = $50,000 under $75,000 | 155 | 24.4% | 6245 | 16.0% |  |
| 4 = $75,000 under $100,000 | 37 | 5.8% | 1636 | 4.2% |  |
| 5 = $100,000 under $200,000 | 4 | 0.6% | 117 | 0.3% |  |
| 6 = $200,000 or more | 0 | 0.0% | 1 | 0.0% |  |
| **Insurance type**** |  |  |  |  |  |
| Commercial | 502 | 79.1% | 31181 | 79.7% | <0.01 |
| Medicaid | 118 | 18.6% | 6090 | 15.6% |  |
| Medicare | 13 | 2.1% | 1541 | 3.9% |  |
| Other | 0 | 0.0% | 46 | 0.1% |  |
| Self-pay | 1 | 0.2% | 258 | 0.7% |  |

SCD, sickle cell disease; T2DM, type 2 diabetes mellitus.

*Based on Zip Code Characteristics: Median Household Income using the American Community Survey (ACS) providing characteristics at the zip code level with the release of the first 5-year product (2005-2009).

** 21 cases missing

| **Supplemental Table 2.** Demographic and clinical characteristics of sickle cell disease patients by genotype, 2008-2019 | | | | | | | | | |
| --- | --- | --- | --- | --- | --- | --- | --- | --- | --- |
|  | **HbSS w/T2DM**  **(n=35)** | | **HbSC**  **w/T2DM**  **(n=15)** | | **HbS-Thalassemia**  **w/T2DM**  **(n=5)** | | **Unknown**  **w/T2DM**  **(n=34)** | | *P* |
|  | n | (%) |  |  |  |  |  |  |  |
| **Patients at risk** | 335 | | 105 | | 20 | | 174 | |  |
| **Crude prevalence (95%CI)** | 10.4% | 7.6-14.2% | 14.3% | 8.7-22.4% | 25.0% | 10.8-47.3% | 19.5% | 14.3-26.1% |  |
| **Standardized prevalence rate** | 14.5% | | 18.9% | | 16.8% | | 21.8% | |  |
| **Age, y** |  |  |  |  |  |  |  |  |  |
| Mean (SD) | 41.2 | 12.3 | 51.0 | 14.3 | 41.2 | 13.0 | 42.3 | 14.1 |  |
| Median (interquartile range) | 42 | 29-51 | 53 | 40-62 | 47 | 34-49 | 44 | 29-55 | 0.13 |
| **Sex** |  |  |  |  |  |  |  |  |  |
| Male | 12 | 34.3% | 3 | 20.0% | 3 | 60.0% | 11 | 32.4% | 0.42 |
| Female | 23 | 65.7% | 12 | 80.0% | 2 | 40.0% | 23 | 67.6% |  |
| **BMI** |  |  |  |  |  |  |  |  |  |
| Mean, SD | 25.8 | 5.3 | 32.2 | 9.0 | 33.1 | 5.1 | 30.0 | 7.8 |  |
| Median (IQR) | 26.5 | 21.5-29.3 | 31.2 | 27.7-36.0 | 30.1 | 29.7-35.8 | 29.2 | 23.7-34.6 | <0.01 |
| **Charlson comorbidity score*** | | | | | | | | | |
| 0 | 2 | 0.0% | 0 | 13.3% | 0 | 0.0% | 0 | 0.0% | 0.24 |
| 1 | 6 | 2.9% | 2 | 40.0% | 1 | 20.0% | 4 | 11.8% |  |
| 2 | 1 | 5.7% | 1 | 6.7% | 2 | 40.0% | 5 | 14.7% |  |
| 3+ | 26 | 5.7% | 12 | 173.3% | 2 | 40.0% | 25 | 73.5% |  |
| **T2DM related microangiopathy** | | | | | | | | | |
| Nephropathy | 17 | 48.6% | 6 | 40.0% | 1 | 20.0% | 16 | 47.1% | 0.65 |
| Peripheral neuropathy | 7 | 20.0% | 4 | 26.7% | 1 | 20.0% | 11 | 32.4% | 0.69 |
| Ophthalmic Complications | 5 | 14.3% | 2 | 13.3% | 1 | 20.0% | 4 | 11.8% | 0.96 |
| **T2DM related macroangiopathy** | | | | | | | | | |
| Peripheral circulatory complications | 5 | 14.3% | 3 | 20.0% | 0 | 0.0% | 8 | 23.5% | 0.54 |
| Foot ulcer | 5 | 14.3% | 2 | 13.3% | 0 | 0.0% | 4 | 11.8% | 0.84 |
| Myocardial infarction | 5 | 14.3% | 3 | 20.0% | 0 | 0.0% | 4 | 11.8% | 0.70 |
| **T2DM related metabolic diabetic complications** | 0 | 0.0% | 0 | 0.0% | 0 | 0.0% | 1 | 2.9% | 0.65 |
| To compare groups, we performed χ^2^ and Wilcoxon rank-sum tests for categorical variables and medians respectively.  *Modified weighted Charlson comorbidity index score (Deyo, et al. *J Clin Epidemiol* 1992) | | | | | | | | | |

BMI, body mass index; T2DM, type 2 diabetes mellitus.

| **Supplemental Table 3.** Relative risk of T2DM among sickle cell disease patients compared to African Americans, adjusted for sex, age BMI, insurance plan and income, 2008-2019 | | | | |
| --- | --- | --- | --- | --- |
| **Characteristics** | Odds ratio | 95% Confidence Interval | | P-value |
| **SCD status** | 1.01 | 0.79 | 1.27 | 0.96 |
| **Male vs. Female** | 0.90 | 0.87 | 0.92 | <0.01 |
| **BMI (every 1 point incremental)** | 1.04 | 1.03 | 1.04 | <0.01 |
| **Age (every 1 year incremental)** | 1.05 | 1.05 | 1.05 | <0.01 |

SCD, sickle cell disease; T2DM, type 2 diabetes mellitus; BMI, body mass index.

* Multivariable logistic regression was used in assessing relative risks for developing T2DM between SCD statuses. Regression model was also adjusted for income (by zip code) and healthcare plan types (data not shown).

| **Supplemental Table 4.** Comparison of glucose levels (mg/dL) by SCD status, adjusted and non-adjusted results, 2008-2019 | | | |
| --- | --- | --- | --- |
| **Characteristics** | **Estimate** | **Standard Error** | **P-value** |
| **Unadjusted** |  |  |  |
| **SCD status** | -27.08 | 4.09 | <0.01 |
| **Adjusted** |  |  |  |
| **SCD status** | -17.30 | 4.38 | <0.01 |
| **Male vs. Female** | 8.71 | 5.04 | 0.08 |
| **BMI (every 1 point incremental)** | 1.16 | 0.24 | <0.01 |
| **Age (every 1 year incremental)** | 0.38 | 0.16 | 0.02 |

SCD, sickle cell disease; T2DM, type 2 diabetes mellitus; BMI, body mass index.

*We used linear marginal multilevel model to provide population-level difference in glucose accounting with within subject correlations. Timing of test were not considered, but we have excluded all glucose tests performed in the inpatient and ER settings.
